# 520. Pharmacokinetic and Safety Phase 1 Study and Microneutralization Assay Results with BRII-196/BRII-198, a Novel Antibody Cocktail Active Against a Wide Range of SARS-CoV-2 Variants

**DOI:** 10.1093/ofid/ofab466.719

**Published:** 2021-12-04

**Authors:** David A Margolis, Fujie Zhang, Xiaohua Hao, Yanyan Li, Mingming Wang, Chunming Li, Yao Zhang, Ji Ma, Yun Ji, Qing Zhu

**Affiliations:** 1 Brii Biosciences, Chapel Hill, NC; 2 Beijing Ditan Hospital, Beijing, China

## Abstract

**Background:**

BRII-196 and BRII-198 are human monoclonal antibodies (mAb) with an extended half-life targeting distinct epitopes of the spike protein on SARS-CoV-2. Mutations in these epitope regions are continuously emerging, potentially conferring resistance to COVID-19 therapeutics in development. Individual phase I studies showed that BRII-196 or BRII-198 alone were safe and well tolerated in healthy subjects. The BRII-196 and BRII-198 cocktail is currently under evaluation in Phase 2/3 studies for the treatment of COVID-19.

**Methods:**

Preclinical study: BRII-196 and BRII-198 were evaluated in the microneutralization assay using pseudo-viruses encoding mutations identified in the spike protein of a panel of SARS-CoV-2 variants of concerns, including strains originating in UK, SA, BR, CA, and India. The fold-change in neutralization IC_50_ titers relative to wild-type virus was calculated. Phase 1 study: healthy adults received sequential IV BRII-196 and BRII-198 (n=9) or placebo (n=3); and were followed for 180 days. Two dose levels (750mg/750mg and 1500mg/1500mg) were evaluated for safety, pharmacokinetics and immunogenicity. Interim analysis results are presented.

**Results:**

Preclinical: BRII-196 and BRII-198 exhibited neutralizing activity against pseudo-virus variants that contained spike mutations of a panel of variants including B.1.1.7 (UK), B.1.351(SA), P.1(BR), B.1.427/429 (CA), B.1.526 (NY), and B.1.617 (IN), comparable to that against wild-type virus. Phase I study: BRII-196 plus BRII-198 was well tolerated with no dose-limiting adverse events (AEs), deaths, serious adverse events, or infusion reactions. The majority of AEs were isolated asymptomatic grade 1-2 laboratory abnormalities. (Table 1). Each mAb displayed pharmacokinetic characteristics expected of extended half-life YTE-antibodies.

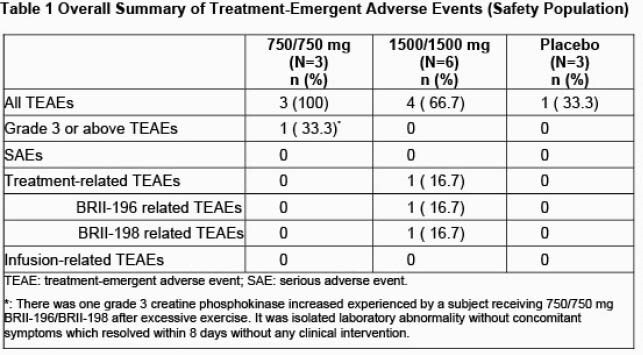

**Conclusion:**

The BRII-196 and BRII-198 cocktail was well-tolerated, and maintains neutralization against currently reported circulating variants of concern. These preclinical and clinical results support further development of BRII-196 and BRII-198 as a therapeutic or prophylactic option for SARS-CoV-2.

**Disclosures:**

**David A. Margolis, MD MPH**, Brii Biosciences (Employee) **Yao Zhang, MD**, Brii Biosciences (Employee) **Yun Ji, PhD**, Brii Biosciences (Employee, Shareholder)

